# High-efficiency cellulosic biocatalysts constructed via *Pichia pastoris* surface display

**DOI:** 10.1186/s13568-026-02095-5

**Published:** 2026-07-29

**Authors:** Lantian Wang, Leijia He, Mengxi Xie, Jingyuan Huang, Chunyi Wang, Shuai Zhang, Xiaoguang Yan, Lin Yuan

**Affiliations:** College of Life Sciences, Inner Mongolia University, Hohhot, 010070 People’s Republic of China

**Keywords:** *Pichia pastoris*, Surface display, Cellulose, Whole-cell catalyst, Fermentation optimization

## Abstract

The high cost and limited stability of cellulases pose significant barriers to their industrial application in biomass conversion. Yeast surface display technology offers a promising approach for constructing reusable whole-cell catalysts; however, the compatibility between different anchor proteins and specific cellulase components remains unclear. In this study, *Pichia pastoris* X33 was used as the host strain to construct nine recombinant strains by fusing three anchor proteins (PIR1, SED1, AGA1) with endoglucanase (EG), cellobiohydrolase (CBH), and beta-glucosidase (BGL), respectively. Notably, PIR1 demonstrated markedly higher display efficiency for all three enzymes compared to SED1 and AGA1. Notably, the EG-PIR1 combination achieved the highest display efficiency, reaching 91.5% as quantified by flow cytometry. Following fermentation optimization, enzyme activity surged to 0.594 U/mL, marking a 42.2% improvement over pre-optimization levels. Furthermore, the surface-displayed enzymes retained over 75% relative activity after six consecutive cycles of reuse. This study quantitatively elucidates the adaptation patterns between anchor proteins and cellulase components, providing a theoretical foundation and technical pathway for the rational design of highly efficient whole-cell biocatalysts.

**Supplementary Information:**

The online version contains supplementary material available at https://doi.org/10.1186/s13568-026-02095-5.

## Introduction

Lignocellulosic biomass, the most widely distributed and abundant renewable resource, is a vital source of future energy and chemical feedstocks, and its valorization is a key step in realizing the bioeconomy (Dharmaraja et al. 2023). Biological methods that utilize specific microorganisms (primarily white-rot and brown-rot fungi) or the enzyme systems they secrete can efficiently degrade cellulose under mild conditions. These methods offer comprehensive advantages, including high specificity, low energy consumption, no inhibitory by-products, and environmental compatibility (Areeshi 2022), and are considered the most favorable route for the resource utilization of cellulose.

Cellulases hydrolyze *β*-1,4-glycosidic bonds in cellulose, and their complete action requires the synergistic activity of three components: endoglucanase (EG), cellobiohydrolase (CBH), and *β*-glucosidase (BGL) (Sharma et al. 2016). Despite their considerable theoretical potential, the large-scale industrial application of cellulases remains constrained by several critical challenges, including high production costs (Ferreira et al. 2021), insufficient enzymatic activity (Westh et al. 2025), poor operational stability, limited catalytic efficiency, and the inherent difficulties associated with industrial fermentation processes.

To overcome these limitations inherent to free enzyme systems, enzyme immobilization technologies have emerged and advanced rapidly as a viable alternative strategy. Conventional immobilization approaches (including physical adsorption, covalent binding, entrapment, and cross-linking) confine enzyme molecules within or onto defined support matrices. These methods have been demonstrated to substantially enhance operational stability and reusability, streamline downstream product separation and purification, and reduce overall production costs, thereby opening new avenues for innovation in enzyme immobilization (Zhang et al. 2025).

Cell surface display technology has emerged as a novel in situ immobilization strategy with significant promise for addressing the aforementioned bottlenecks. In this approach, genetic engineering is employed to fuse the gene encoding the target exogenous protein with that of a specific anchor protein, generating a recombinant expression construct. Upon introduction into the host cell, the resulting fusion protein is directed to the extracellular space by a signal peptide, where it is immobilized on the surface of living cells through covalent or non-covalent interactions between the anchor protein and cell wall or cell membrane components (Wu et al. 2008). The displayed fusion proteins are capable of maintaining relatively independent spatial conformations and biological activities, while the host cells themselves can serve as whole-cell catalysts to participate directly in the reaction (Kuroda and Ueda 2013). In contrast to conventional carrier-based immobilization methods, surface display technology achieves in situ enzyme immobilization, the anchoring process occurs concurrently with protein expression in living cells, eliminating the need for elaborate purification steps. Moreover, the continuous renewal of surface-displayed enzyme proteins by viable cells, combined with the capacity of host cells to function directly as whole-cell catalysts, further distinguishes this approach from traditional immobilization strategies (Wang et al. 2024). As demonstrated by Anandharaj et al. (Anandharaj et al. 2020), cellulases have been successfully displayed on the cell surface, enabling high-efficiency ethanol production. In a related study, Wang et al. (Wang et al. 2026) reported the successful display of the bacterial fimbrial adhesin FimH on the surface of *Saccharomyces cerevisiae*, which significantly enhanced the biofilm-forming capacity of the yeast.

Among the existing studies, *S. cerevisiae* has been the most widely employed host for cell surface display, whereas the utilization of alternative yeast hosts remains relatively underexplored (Lozančić et al. 2019). In this study, *Pichia pastoris* was selected as the host organism based primarily on its distinct advantages, suitability for high-density fermentation (Sarsaiya et al. 2019), and the availability of strong inducible promoters (Vogl 2022). In addition, three representative anchor proteins (SED1, AGA1, and PIR1) were selected. By systematically comparing the display efficiencies of these three anchor proteins across three cellulases, this study aims to elucidate the influence of anchor protein type on display performance, thereby providing a rational basis for the informed selection and optimized design of anchor proteins in future applications. Furthermore, it establishes a basis for the rational design of efficient yeast surface display systems and the construction of robust whole-cell biocatalysts for cellulose degradation. Ultimately, this approach offers a promising technological route toward the high-value valorization of lignocellulosic biomass.

## Materials and methods

### Strains and plasmids

The *P. pastoris* X33 strain, *Escherichia coli* strain DH5*α* and the plasmid pPICZ*α*A used in this study were obtained from the laboratory collection. Codon-optimized synthesis of the cellulase genes was performed by Sangon Biotech (Shanghai) Co., Ltd. Primer synthesis and DNA sequencing were carried out by Beijing Liuhe BGI Technology Co., Ltd.

### Medium

The culture medium formula is as follows:

LB medium: 5 g/L yeast extract, 10 g/L tryptone, 10 g/L NaCl (add 2.0% agar powder to the solid medium); use at natural pH after sterilization at 121 °C for 20 min. For media containing antibiotics, once the temperature has dropped below 55 °C, add a stock solution of bleomycin to a final concentration of 50 μg/mL in a laminar flow hood.

YPD medium: 10 g/L yeast extract, 20 g/L peptone, 20 g/L glucose (add 2.0% agar powder to the solid medium), adjust the pH to 7.0–7.2, and use after sterilization at 115 °C for 30 min. For medium containing antibiotics, after the temperature has dropped below 55 °C, add bleomycin stock solution on a laminar flow hood to a final concentration of 100 μg/mL.

BMGY liquid medium: 10 g/L yeast extract, 20 g/L peptone, K_2_HPO_4_ 3 g/L,KH_2_PO_4_ 11.8 g/L, add distilled water to 890 mL; sterilize at 121 °C for 20 min; then, after the temperature has dropped to 55 °C, add 100 mL of 10 × YNB solution (134 g/L), 1 mL of 500 × biotin (4 × 10^−4^ g/L), and 10 mL of glycerol on a laminar flow hood.

BMMY liquid medium: 10 g/L yeast extract, 20 g/L peptone, K_2_HPO_4_ 3 g/L, KH_2_PO_4_ 11.8 g/L, add distilled water to 895 mL, sterilize at 121 °C for 20 min, then, after the temperature has dropped to 55 °C, add 100 mL of 10 × YNB solution (134 g/L), 1 mL of 500 × biotin (4 × 10^−4^ g/L), and 5 mL of methanol on a laminar flow hood.

### Methods

#### Acquisition of the target gene and design of the expression vector

Based on a review of the literature, three cellulase genes reported to exhibit relatively high expression levels were selected: the EG gene (GenBank ID: KU751779.1) from *Trichoderma reesei*, the CBH gene (GenBank ID: XM_006962518.1) from *Trichoderma viride*, and the BGL gene (GenBank ID: XM_020202409.1) from *Aspergillus aculeatus*. The signal peptide sequence of each gene was predicted and subsequently removed using the SignalP-6.0 online tool. A flexible linker peptide (G_4_S)_3_ was introduced at the N-terminus, flanked upstream by an *Eco*RI restriction site and downstream by a *Not*I restriction site at the C-terminus. Immediately downstream of the *Not*I site, a sequence encoding a 6 × His tag was added to facilitate downstream protein purification and immunofluorescence detection. The designed sequences were synthesized by Sangon Biotech (Shanghai) Co., Ltd.

Anchor protein genes were selected from the *S. cerevisiae* genome maintained in the laboratory collection. These included the GPI-type anchor protein genes SED1 (GenBank ID: AB530985.1) and AGA1 (GenBank ID: NM_001183221.1), as well as the PIR-type anchor protein gene PIR1 (GenBank ID: XM_056229274.1). All optimized gene sequences are provided in the supplementary materials.

Each anchor protein gene was individually fused with the cellulase genes to generate fusion constructs, which were subsequently ligated into pPICZ*α*A to yield the recombinant expression vectors. During the fusion process, the original *Eco*RI site introduced at the N‑terminus was disrupted upon seamless cloning, whereas the *Xho*I site (located upstream of *Eco*RI) and the introduced *Not*I site remained available for subsequent plasmid verification. Therefore, *Xho*I and *Not*I double digestion was used to verify the correct assembly of each recombinant plasmid.

#### Construction, screening and identification of recombinant plasmids

Using the anchor protein gene sequences maintained in the laboratory collection as templates, seamless cloning primers were designed according to the corresponding gene sequences, PCR amplification was then performed to generate anchor protein gene fragments flanked by vector-specific homologous arms. The primer sequence is provided in Table S1. The pPICZ*α*A vector harboring the cellulase gene insert was linearized by single digestion with *Eco*RI, yielding the vector backbone containing the cellulase gene.

The anchor protein gene fragments and the linearized vector backbone harboring the cellulase gene were ligated using a homologous recombination-based seamless cloning kit (*pEASY*®-Basic Seamless Cloning and Assembly Kit, TransGen, Beijing, China). The resulting constructs were transformed into *E.coli* DH5*α* competent cells via heat shock, and recombinant plasmids were obtained following positive clone selection.

#### Construction, screening and characterisation of recombinant *P. pastoris* strains

The recombinant plasmids pPICZ*α*A-EG-PIR1, pPICZ*α*A-EG-SED1, pPICZ*α*A-EG-AGA1, pPICZ*α*A-CBH-PIR1, pPICZ*α*A-CBH-SED1, pPICZ*α*A-CBH-AGA1, pPICZ*α*A-BGL-PIR1, pPICZ*α*A-BGL-SED1, and pPICZ*α*A-BGL-AGA1 were linearized by digestion with *Pme*I and subsequently introduced into *P. pastoris* X33 competent cells via electroporation. Positive transformants were selected on YPD solid medium supplemented with Zeocin. Genomic DNA was then extracted from the transformants and subjected to PCR amplification and sequencing for verification. The resulting recombinant *P. pastoris* strains were designated X33-EG-PIR1, X33-EG-SED1, X33-EG-AGA1, X33-CBH-PIR1, X33-CBH-SED1, X33-CBH-AGA1, X33-BGL-PIR1, X33-BGL-SED1, and X33-BGL-AGA1.

#### Determination of surface-displayed enzyme activity

Preparation of the whole-cell biocatalyst: Cultures of methanol-induced recombinant yeast were centrifuged, and the resulting cell pellets were washed with citrate buffer to remove residual medium. The washed cells were then resuspended in the same buffer and used directly as whole-cell biocatalysts for subsequent enzyme activity assays.

Enzyme activity assay: Enzyme activity was determined using the DNS (3,5-dinitrosalicylic acid) colorimetric method, as described below.

Determination of EG activity: A 2 mL aliquot of 1% (*W/V*) carboxymethylcellulose sodium (CMC-Na) dissolved in 50 mM citrate buffer (pH 5.0) was pre-incubated at 50 °C for 3 min. The whole-cell biocatalyst suspension was then added, and the reaction was allowed to proceed at 50 °C for 1 h. The reaction was terminated by the addition of 2 mL of DNS reagent, followed by heating in a boiling water bath for 10 min. After rapid cooling, the volume was adjusted to 15 mL with distilled water, and the absorbance was measured at 540 nm. One unit of EG activity was defined as the amount of enzyme required to liberate 1 μmol of reducing sugar per minute under the assay conditions.

Determination of CBH and BGL activities: For CBH activity assays, 1% (*W/V*) microcrystalline cellulose was used as the substrate, whereas for BGL activity assays, 1% (*W/V*) salicin was employed. All other assay procedures were identical to those described for EG.

After determination, the standard curve (y = 9.0045x, R^2^ = 0.9997) formula for measuring enzyme activity using the DNS method.

Enzyme activity was calculated using the following formula:

$$ \begin{gathered} {\mathrm{Enzyme}}\,{\mathrm{activity}}\left( {{\mathrm{U}}/{\mathrm{mL}}} \right) = \,\left( {{\mathrm{OD}}_{{{\mathrm{exp}}}} - {\mathrm{OD}}_{{{\mathrm{control}}}} } \right) \hfill \\ \quad \quad \quad \times {\mathrm{1}}0^{3} /\left( {{\mathrm{9}}.00{\mathrm{45}} \times {\mathrm{18}}0.{\mathrm{16}} \times {\mathrm{t}} \times {\mathrm{V}}} \right) \hfill \\ \end{gathered} $$

where OD_exp_ and OD_control_ represent the absorbance values of the experimental and control groups, respectively; t is the reaction time (min); and V is the volume of the enzyme solution (mL).

#### Optimisation of recombinant *P. pastoris* culture conditions

To enhance the enzymatic activity of the cellulase displayed on the surface of recombinant *P. pastoris* strains, key culture conditions during fermentation were systematically optimized. Fermentation parameters were optimized using both single-factor experiments and orthogonal array design. The single-factor experiments evaluated the following parameters: induction time (0–192 h), initial medium pH (4.5–7.5), methanol supplementation concentration (0.2–0.8%, supplemented at 12 h intervals), culture temperature (26–34 °C), and shaker speed (160–240 r/min).

Subsequently, orthogonal array design was employed to perform multi-factor synergistic optimization of the fermentation conditions. Four factors-initial pH, methanol concentration, culture temperature, and shaker speed-were selected for the orthogonal experiment based on the preceding single-factor analysis. Continuous fermentation of the recombinant *P. pastoris* strains was subsequently conducted under the optimized conditions, and the enzymatic activity of the surface-displayed cellulase was measured to validate the optimization results.

All experiments were performed in triplicate, with enzyme activity serving as the response variable.

#### Determination of the enzymatic properties of the surface-displayed enzyme

One milliliter of the whole-cell biocatalyst suspension was mixed with 1 mL of the corresponding substrate solution and incubated in a 50 °C water bath for 1 h, followed by rapid cooling. After centrifugation of the reaction mixture, the supernatant was collected for enzyme activity measurement (designated as cycle 1). Following the first activity assay, the pelleted cells were washed twice with PBS buffer and subsequently resuspended in an equal volume of citrate buffer and fresh substrate solution. The next reaction cycle was then performed under identical conditions. This procedure was repeated for a total of six consecutive cycles, with enzyme activity measured separately after each round.

The enzyme activity measured in cycle 1 was taken as 100%, and the relative activity (%) for each subsequent cycle was calculated using the following formula:

$$ \begin{aligned} {\mathrm{Relative}}\,{\mathrm{activity}}\left( \% \right) = & \,\left( {{\mathrm{Activity}}\,{\mathrm{in}}\,{\mathrm{cycle}}\,{\mathrm{n}}} \right./ \\ & \quad \left. {{\mathrm{Activity}}\,{\mathrm{in}}\,{\mathrm{cycle}}\,{1}} \right) \times {1}00\% \\ \end{aligned} $$

All experiments were performed in triplicate, and the retention of enzyme activity was plotted as a function of reaction cycle.

#### Validation of surface display efficiency

The methods for immunofluorescence observation and flow cytometry analysis were as follows:

Cell cultures induced for 144 h were centrifuged to collect the cells, which were subsequently washed twice by resuspension in pre-chilled PBS buffer. The washed cells were resuspended in PBS-BSA buffer, and anti-His tag mouse monoclonal antibody (6 × His, His-tag monoclonal antibody, Proteintech, Wuhan, China) was added at a dilution of 1:1000. Following incubation at 4 °C for 1–2 h, the cells were collected by centrifugation and washed twice with pre-chilled PBS buffer. The cells were then resuspended in PBS-BSA buffer, and FITC-conjugated Goat Anti-Mouse IgG(H + L) secondary antibody (Proteintech, Wuhan, China) was added at a dilution of 1:200. The samples were incubated at 4 °C for 3–4 h in the dark. After incubation, the cells were collected by centrifugation and washed twice with pre-chilled PBS buffer. Finally, the cells were resuspended in PBS buffer to a final concentration of approximately 10^6^ cells/mL. Slides were prepared for observation under a fluorescence microscope, and the remaining samples were subjected to flow cytometry analysis.

## Results

### Construction of the *P. pastoris* surface display system

To verify the correct assembly of the recombinant plasmids, the constructed plasmids pPICZ*α*A-EG-PIR1, pPICZ*α*A-EG-SED1, pPICZA-EG-AGA1, pPICZ*α*A-CBH-PIR1, pPICZ*α*A-CBH-SED1, pPICZ*α*A-CBH-AGA1, pPICZ*α*A-BGL-PIR1, pPICZ*α*A-BGL-SED1, and pPICZ*α*A-BGL-AGA1 were subjected to double digestion with *Xho*I and *Not*I. The digested products were then analyzed by 1% agarose gel electrophoresis; the expected fragment sizes confirmed the successful insertion of each fusion gene. Single colonies selected on YPD agar plates supplemented with Zeocin were picked and cultured for expansion. Genomic DNA was extracted from the recombinant *P. pastoris* strains and subjected to PCR amplification using the universal primers. The PCR products were subsequently verified by sequencing, which revealed 100% identity with the expected sequences. These results confirmed that the recombinant plasmids had been successfully integrated into the genome of *P. pastoris* X33, demonstrating the successful construction of the recombinant strains.

### Determination of surface-displayed enzyme activity

Among the nine recombinant strains, X33-EG-PIR1 exhibited the highest enzymatic activity (0.418 ± 0.012 U/mL). For each of the three cellulases (EG, CBH, and BGL), the PIR1 anchor protein consistently conferred higher enzymatic activity than SED1 and AGA1 (Table 1).

**Table 1:** Enzyme activity assay results of surface-displayed enzymes (Unit: U/mL)

Gene name	PIR1	SED1	AGA1
EG	0.418 ± 0.012	0.314 ± 0.019	0.287 ± 0.009
CBH	0.170 ± 0.015	0.142 ± 0.012	0.124 ± 0.008
BGL	0.261 ± 0.010	0.227 ± 0.002	0.192 ± 0.003

Data shown are mean ± SD (n = 3)

### Optimisation of fermentation conditions for recombinant *P. pastoris*

Multi-factor synergistic optimization of the fermentation conditions for the recombinant *P. pastoris* strains was performed using an orthogonal array design. Four key factors were investigated: initial medium pH, methanol concentration, fermentation temperature, and shaker speed. The recombinant strain X33-EG-PIR, which exhibited the highest enzymatic activity among the nine constructed strains, was selected for the orthogonal optimization experiment. The remaining eight strains were subsequently fermented under the optimized conditions to validate the general applicability of the optimization results; a comparison of enzyme activities before and after optimization for all nine strains is summarized in Table 2. Both range analysis and analysis of variance revealed that the four factors influenced the enzymatic activity of the surface-displayed cellulase in the following order of significance: initial medium pH > methanol concentration > fermentation temperature > shaker speed. Furthermore, initial medium pH and methanol concentration exerted a highly significant effect on enzyme activity (*P* < 0.01), whereas fermentation temperature exhibited a significant effect (*P* < 0.05). The optimized fermentation conditions were determined as follows: initial medium pH, 5.0 (6.0 for BGL); methanol supplementation, 0.7% added at 12 h intervals; fermentation temperature, 28 °C (30 °C for BGL); and shaker speed, 200 r/min.

**Table 2:** Comparison of enzyme activity determination before and after optimisation of fermentation conditions (Units: U/mL)

Strain name	Enzyme activity before optimisation	Enzyme activity after optimisation	Percentage increase	Significance
X33-EG-PIR1	0.418 ± 0.012	0.594 ± 0.003	42.2%	***
X33-EG-SED1	0.314 ± 0.019	0.445 ± 0.011	41.8%	***
X33-EG-AGA1	0.287 ± 0.009	0.401 ± 0.003	39.4%	**
X33-CBH-PIR1	0.170 ± 0.015	0.223 ± 0.006	31.6%	**
X33-CBH-SED1	0.142 ± 0.012	0.186 ± 0.004	30.7%	*
X33-CBH-AGA1	0.124 ± 0.008	0.159 ± 0.002	28.1%	**
X33-BGL-PIR1	0.261 ± 0.010	0.343 ± 0.004	31.3%	**
X33-BGL-SED1	0.227 ± 0.002	0.294 ± 0.003	29.2%	*
X33-BGL-AGA1	0.192 ± 0.003	0.246 ± 0.004	28.0%	*

Data shown are mean ± SD (n = 3). Statistical analysis was performed by the *t*-test (^*^p < 0.05, ^**^p < 0.01, ^***^p < 0.001)

### Study of the enzymatic properties of surface-displayed enzymes

**Fig. 1:**
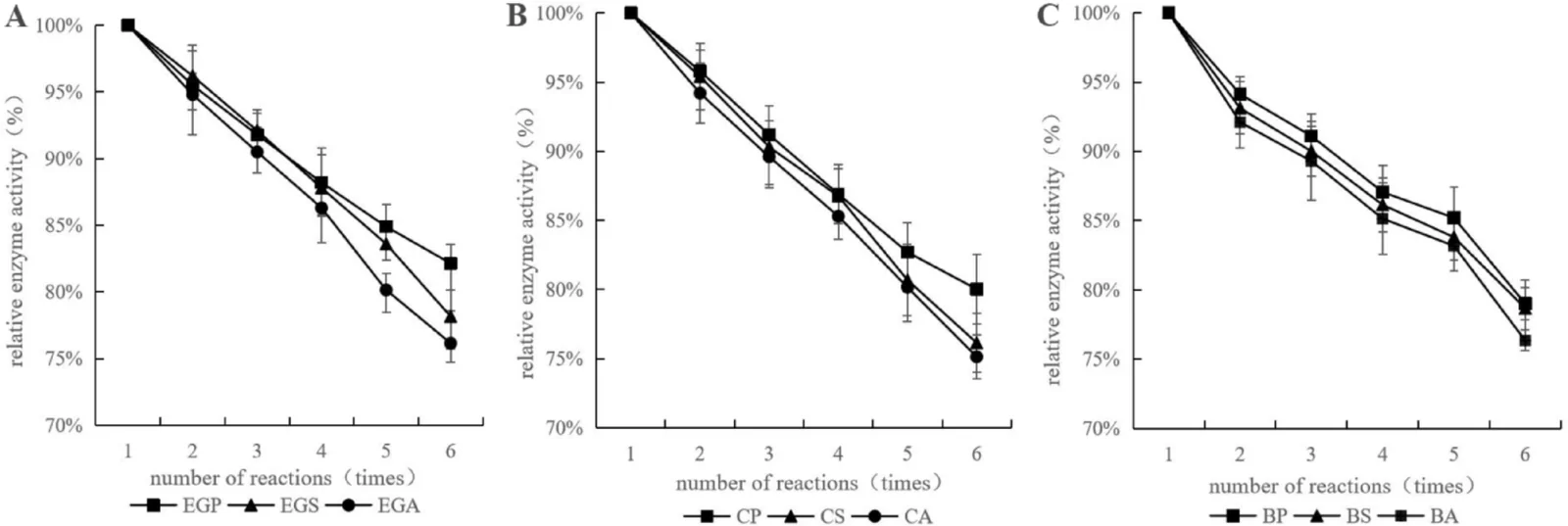
Reusability assay of surface-displayed enzymes. **A** Measurement of the reusability of surface-displayed EG; **B** Measurement of the reusability of surface-displayed CBH; **C** Measurement of the reusability of surface-displayed BGL

To evaluate the enzymatic properties of the surface-displayed cellulases, the reusability of the whole-cell biocatalysts was assessed. Following each round of enzyme activity measurement, the cell pellets were recovered by centrifugation, washed, and subsequently resuspended in fresh substrate solution for the next reaction cycle. This procedure was repeated to simulate the repeated addition of cellulase in industrial applications. As shown in Fig. 1, the surface-displayed cellulases exhibited excellent reusability, with relative activities remaining above 75% after six consecutive cycles of recovery and reuse. Notably, the X33-EG-PIR1 and X33-CBH-PIR1 strains retained 82.14% and 80.03% of their initial activities, respectively, whereas the remaining strains retained between 75.14% and 79.01% after six cycles. These results demonstrate that the cellulases were successfully and stably displayed on the surface of the recombinant *P. pastoris* cells, exhibiting robust operational stability. This provides a promising foundation for potential industrial applications.

### Immunofluorescence observation

**Fig. 2:**
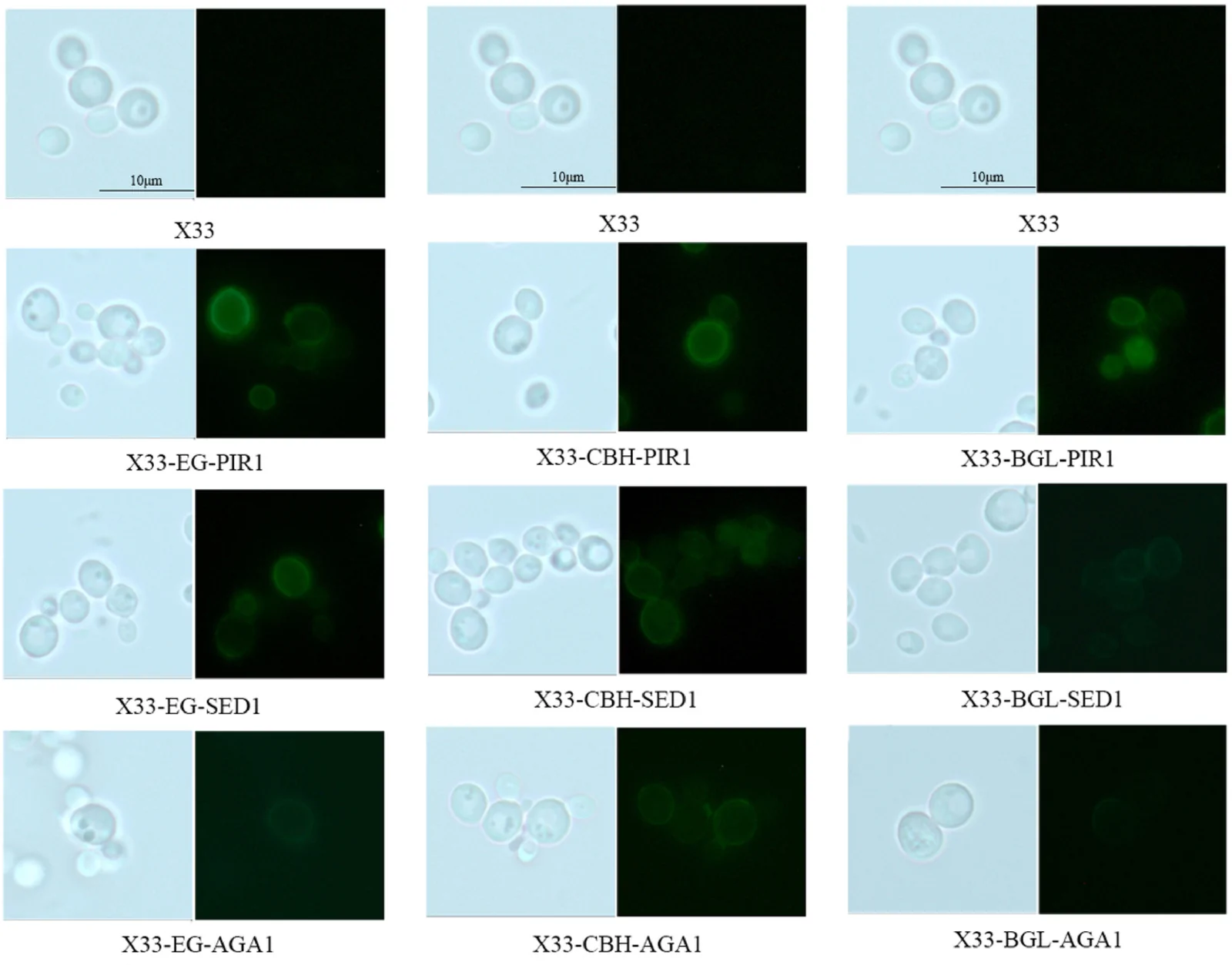
Image of immunofluorescence observation of recombinant *P. pastoris* surface-displayed cellulase

To determine the subcellular localization of the surface-displayed cellulases, the induced recombinant yeast strains were subjected to immunofluorescence staining and subsequently examined under a fluorescence microscope. As shown in Fig. 2, the parental X33 strain (negative control) exhibited no detectable fluorescence, whereas all recombinant strains displayed distinct fluorescent signals, confirming the successful display of cellulases on the cell surface. Notably, strains X33-EG-PIR1 and X33-BGL-PIR1 exhibited the highest fluorescence intensities, while strains X33-CBH-AGA1 and X33-BGL-AGA1 showed the weakest signals. These observations were consistent with the enzyme activity assay results.

### Flow cytometry analysis

**Fig. 3:**
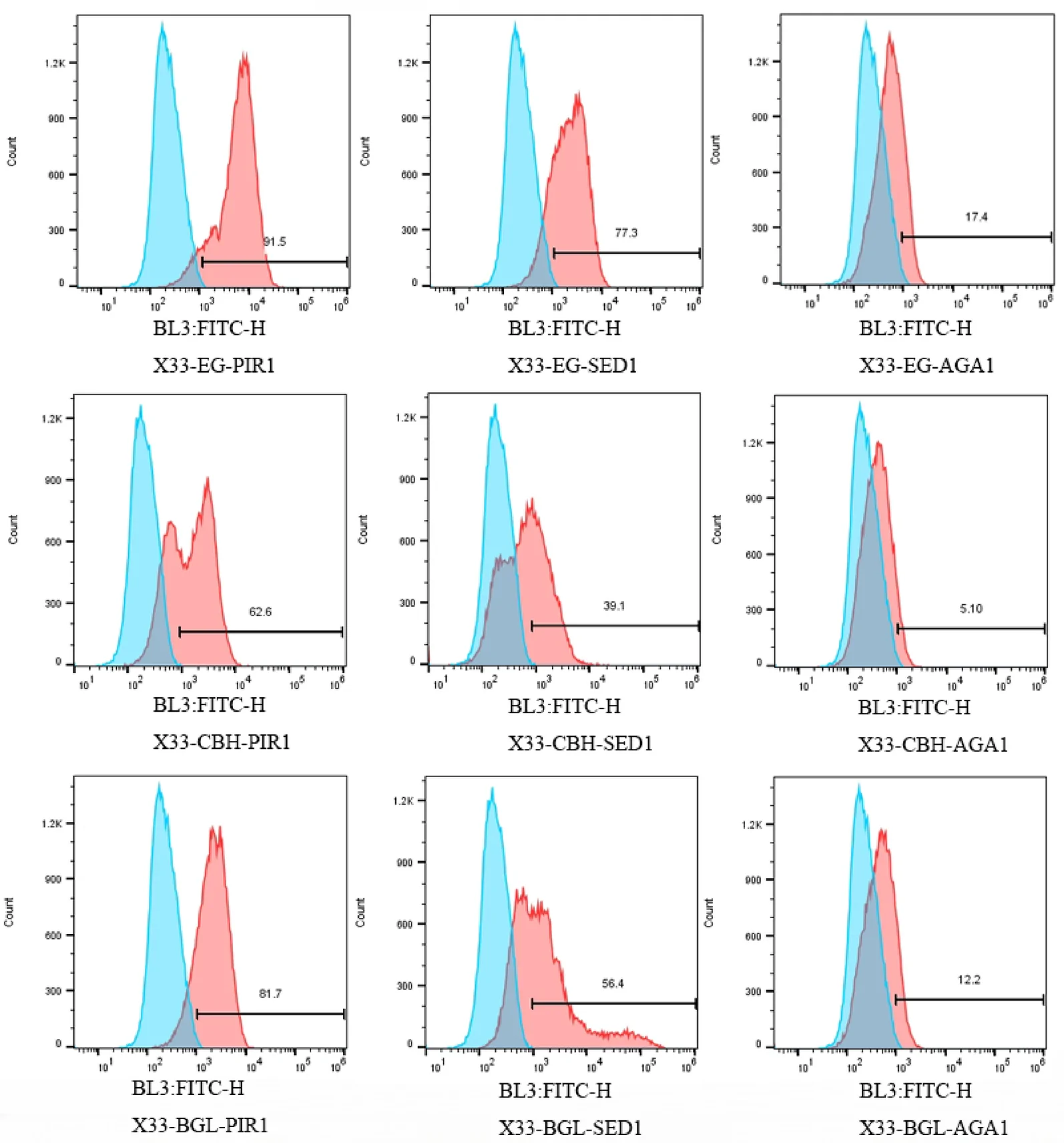
Flow cytometry analysis results

To quantify the surface display efficiency of the recombinant yeast strains, the recombinant *P. pastoris* strains were analyzed by flow cytometry. Following induction, the fermentation cultures were processed according to the procedure described in Section Validation of surface display efficiency, and 1 mL of the resulting cell suspension was transferred to a specialized flow cytometry tube for analysis. The parental *P. pastoris* X33 strain was used as a negative control. A total of 50,000 events were collected for each sample, and all measurements were performed in triplicate. As shown in Fig. 3, the blue peaks represent the fluorescence profile of the negative control (parental X33 strain), whereas the red peaks correspond to the fluorescence signals of the recombinant strains displaying cellulases on their surface. A distinct rightward shift in fluorescence intensity was observed for all recombinant strains, confirming the successful display of cellulases on the surface of the recombinant *P. pastoris* cells. Surface display efficiency was quantified as the percentage of the fluorescence peak area exceeding the overlap with the negative control (as indicated by the numerical values on the red peaks in Fig. 3). The display efficiencies, ranked from highest to lowest, were as follows: X33-EG-PIR1 (91.5%), X33-BGL-PIR1 (81.7%), X33-EG-SED1 (77.3%), X33-CBH-PIR1 (62.6%), X33-BGL-SED1 (56.4%), X33-CBH-SED1 (39.1%), X33-EG-AGA1 (17.4%), X33-BGL-AGA1 (12.2%), X33-CBH-AGA1 (5.1%). These quantitative results were largely consistent with the immunofluorescence observations. Notably, the PIR1 anchor protein conferred markedly higher display efficiency than either SED1 or AGA1, suggesting that PIR1 is particularly well-suited for cellulase surface display systems. Moreover, the EG-PIR1 combination achieved the highest display efficiency among all constructs tested in this study.

## Discussion

This study represents the first systematic comparison of three anchor proteins (PIR1, SED1, AGA1) for the surface display of three distinct cellulase components (EG, CBH, and BGL) in *P. pastoris*. The results demonstrated that PIR1 conferred superior display efficiency relative to both SED1 and AGA1 across all three enzyme types (Yang et al. 2020). Notably, the EG–PIR1 combination achieved the highest display efficiency (91.5%) and an enzymatic activity of 0.594 U/mL. These findings provide a valuable foundation for the rational selection of anchor proteins in the design of yeast surface display systems. The pronounced efficacy of PIR1 may be attributed to its unique anchoring mechanism (Li et al. 2024). PIR1 belongs to the protein with internal repeats family of anchor proteins, which are covalently attached to the cell wall via direct ester bond formation between their N-terminal repetitive sequences and *β*-1,3-glucan (Sumita et al. 2005). This mechanism circumvents the complex endoplasmic reticulum–Golgi processing and subsequent membrane-to-cell wall transfer steps characteristic of GPI-type anchor proteins, such as SED1 and AGA1 (Kapteyn et al. 1999). This direct and simplified anchoring mode not only enhances display stability but also minimizes potential interference with the spatial conformation of the displayed fusion protein (Abe et al. 2003; Hossain et al. 2019, Martinić Cezar et al. 2023).

SED1 is a GPI-anchored protein that predominantly localizes fusion proteins to the outer cell wall surface (Yang et al. 2019). AGA1 is the anchoring subunit of the *α*-agglutinin system; it undergoes extensive O-glycosylation and is covalently linked to the cell wall via a C-terminal GPI anchor (Lipke and Kurjan 1992). PIR1 belongs to the PIR-type anchor protein family (Alvarado et al. 2025) and is unique in that it forms covalent ester bonds with *β*-1,3-glucan in the cell wall through internal repeat sequences. PIR1 is currently the only known GPI-independent protein anchoring system, characterized by a relatively simple structure and a straightforward anchoring mechanism. All three anchor proteins have been independently validated for heterologous protein surface display in *P. pastoris*, and they represent two distinct anchoring types, GPI-anchored (SED1 and AGA1) and PIR-type (PIR1) exhibiting marked differences in anchoring mode and structural architecture (Andreu and Del Olmo 2018). The favorable reusability observed in this study indicates that the covalent linkage mechanism driven by PIR-type anchor proteins confers enhanced anchoring strength upon displayed enzymes.

It is noteworthy that although AGA1 has been reported to mediate efficient display in *S. cerevisiae* (Mei et al. 2017), it exhibited considerably lower efficiency in the *P. pastoris* system employed in this study (Luo et al. 2026). This discrepancy suggests that the performance of anchor proteins is host-specific and may be attributable to structural differences in the cell wall architecture among distinct yeast species (Jofre et al. 2024). Quantitative flow cytometric analysis revealed a positive correlation between display efficiency and enzymatic activity, underscoring that enhanced display efficiency represents an effective strategy for the development of whole-cell biocatalysts with elevated enzyme activity. Hossain et al. (Hossain et al. 2019) further demonstrated that xylose reductase displayed via the PIR4 anchor protein exhibited superior thermal and pH stability compared with its GPI-anchored counterpart, while suffering less reduction in catalytic activity. Optimization of fermentation conditions demonstrated that both initial pH and methanol concentration exerted highly significant effects on enzyme activity (*P* < 0.01) (Liu et al. 2025). Moreover, the optimal initial pH differed between strains displaying EG/CBH and those displaying BGL. Reusability assays further showed that the relative enzyme activity remained above 75% after six consecutive reaction cycles, confirming the robustness of PIR1-mediated anchoring and supporting the feasibility of these whole-cell biocatalysts for industrial batch applications. Microscopic observation revealed that the recovered cells remained morphologically intact, with no visible debris. Furthermore, the activity declined progressively over six cycles rather than dropping abruptly, which would be expected if cell lysis were the dominant factor. These observations suggest that cell lysis would not the primary cause of the observed decrease in activity. Nevertheless, the direct quantitation of cell viability through CFU plate counting would provide more definitive evidence in the further study.

In contrast to free enzymes, which cannot be recovered, our whole-cell catalysts are easily recycled by centrifugation, offering a significant cost advantage. Compared with other immobilization methods, which typically retain 50–70% of initial activity after six cycles (Li et al. 2019), our surface-displayed cellulases retained > 75% activity (Li et al. 2022), indicating that the robust covalent anchoring of PIR1 and the intact yeast cell structure provide effective enzyme protection. Notably, our approach requires no chemical reagents or carrier materials, making it simpler and greener. Several studies have successfully displayed various proteins (lipases, xylose reductases, glycosyltransferases) using PIR family anchors in both *S. cerevisiae* and *P. pastoris* (Abe et al. 2003; Hossain et al. 2019). Our study further demonstrates that PIR1 is superior to GPI-anchored proteins for displaying three cellulases of different molecular weights and origins. Given its direct covalent anchoring, simple domain organization, and minimal conformational interference, PIR1 appears broadly applicable, especially for industrial enzymes where activity must be preserved. However, for proteins relying on specific membrane localization or glycosylation, GPI-anchored proteins may still be advantageous. Future work will include evaluation of the PIR1-based display system on native crystalline cellulose substrates such as Avicel, as well as construction of a multi-enzyme co-display system to investigate the synergistic degradation of cellulose by the three cellulase components.

Nevertheless, considerable challenges remain before surface-displayed cellulases can be deployed for practical industrial applications, as the enzymatic activity of whole cell biocatalysts currently lags behind that of their free enzyme counterparts (Inokuma et al. 2023). Future investigations may therefore be directed toward the following avenues: (1) construction of a multi enzyme co display system to optimize the stoichiometric ratio of the three cellulase components on the cell surface (Phienluphon et al. 2024), thereby mimicking the synergistic action of natural cellulolytic enzyme systems; (2) extension of the PIR1 based anchoring system to other industrially relevant enzymes to evaluate its general applicability; (3) evaluation of whole cell biocatalytic performance and repeated batch fermentation using authentic lignocellulosic biomass as the substrate to assess industrial feasibility; (4) application of protein engineering strategies, such as shortening the linker peptide between the anchor protein and the enzyme or substituting it with a rigid linker, to further enhance the enzymatic activity and catalytic efficiency of the displayed enzymes.

## Supplementary Information

Below is the link to the electronic supplementary material.

Supplementary Material 1

## Data Availability

No datasets were generated or analysed during the current study.
