# Supplementary material for: High-efficiency cellulosic biocatalysts constructed via Pichia pastoris surface display
Source: AMB Express. 2026 Jul 29;16:104. doi: 10.1186/s13568-026-02095-5 (PMC13719967; doi:10.1186/s13568-026-02095-5)
Supplement: Supplementary file 1 [file 13568_2026_2095_MOESM1_ESM.docx]

**Supplementary material**

**High-Efficiency Cellulosic Biocatalysts Constructed via *Pichia pastoris* Surface Display**

Lantian Wang^1^, Leijia He^1^, Mengxi Xie^1^, Jingyuan Huang^1^, Chunyi Wang^1^, Shuai Zhang^1^, Xiaoguang Yan^1^*, Lin Yuan^1^*

^1^ College of Life Sciences, Inner Mongolia University. Hohhot 010070, P.R. China

* Corresponding author E-mail: yuanlin@imu.edu.cn; [yanxiaoguang260@imu.edu.cn](mailto:yanxiaoguang260@imu.edu.cn)

Lin Yuan, Prof.

College of Life Sciences, Inner Mongolia University. Hohhot 010070

Tel: +86-131-7141-9008

Xiaoguang Yan, Dr.

College of Life Sciences, Inner Mongolia University. Hohhot 010070

Tel: +86-185-2644-2385

**Table S1** Primer used in this study

| No. | Primer name | Sequence (5’ to 3’) |
| --- | --- | --- |
| P1 | EG-F | AGAGAGGCTGAAGCTATGGCTTACGCTCCAAAGGATC |
| P2 | EG-R | CAGAACCACCTCCGCCACAGTTCAACAAATCAATAGCT |
| P3 | CBH-F | CTATTGATTTGTTGAACTGTGGCGGAGGTGGTTCTGGCGG |
| P4 | CBH-R | GTTCTGGCGGAGGTGGTTCTGCGGCCGCCAGCTTTC |
| P5 | BGL-F | AGAGAGGCTGAAGCTATGGCTTACGCTCCAAAGGATC |
| P6 | BGL-R | CAGAACCACCTCCGCCACAGTTCAACAAATCAATAGCT |

# The codon-optimized cellulase gene sequences are as follow:

**Endoglucanase gene sequence**

ATGCACCATCATCATCATCATCAACAGACTGTTTGGGGTCAATGTGGTGGAATTGGTTGGTCAGGTCCAACAAATTGTGCTCCTGGATCTGCTTGCTCTACGTTGAACCCATACTACGCTCAATGTATCCCAGGAGCCACTACCATTACTACATCCACACGACCACCATCAGGTCCCACAACTACAACTCGTGCTACTAGTACATCAAGTAGTACTCCTCCCACCTCATCTGGTGTCAGATTCGCAGGAGTGAACATCGCTGGTTTTGATTTTGGTTGCACTACTGACGGTACGTGTGTTACTTCAAAGGTCTATCCTCCTTTAAAGAATTTCACTGGTTCTAACAACTATCCAGATGGCATAGGTCAAATGCAACATTTCGTCAACGACGATGGTATGACCATATTTAGACTGCCAGTTGGCTGGCAATACCTTGTCAACAATAACTTGGGAGGAAATTTGGATTCCACTTCCATTTCAAAGTATGATCAGCTAGTTCAAGGATGTCTTTCTCTTGGTGCTTATTGTATTGTAGATATTCATAATTATGCTCGTTGGAACGGAGGTATAATCGGACAAGGCGGACCAACCAACGCTCAATTTACGTCTCTTTGGTCTCAGTTGGCCAGTAAGTATGCTTCTCAGTCAAGGGTCTGGTTCGGAATCATGAATGAACCTCATGACGTGAATATTAACACCTGGGCCGCAACTGTTCAGGAGGTTGTGACTGCAATTCGAAATGCTGGAGCTACTTCCCAGTTTATTTCATTGCCAGGAAATGACTGGCAGTCAGCTGGAGCATTTATATCCGATGGTTCTGCTGCTGCTTTGTCACAGGTGACTAATCCTGATGGTAGTACTACTAACTTGATTTTCGATGTGCATAAATACCTGGACTCTGACAATAGTGGTACGCATGCAGAGTGTACCACTAATAATATTGACGGTGCTTTTTCACCACTTGCCACTTGGCTGCGTCAAAACAACAGACAAGCTATACTGACTGAGACTGGAGGAGGAAACGTCCAGTCATGCATTCAAGATATGTGCCAACAAATCCAATACCTGAACCAGAATTCTGACGTTTATCTGGGTTACGTTGGATGGGGTGCAGGTTCATTTGATTCCACTTACGTGCTGACGGAGACTCCTACCGGTAGTGGAAATTCATGGACAGATACTTCCTTGGTTTCATCATGTCTTGCCAGGAAATAA

**Cellobiohydrolase gene sequence**

ATGCCTTTGGAAGAAAGACAAGCTTGTTCTTCTGTTTGGGGTCAATGTGGTGGTCAAAATTGGTCTGGTCCAACTTGTTGTGCTTCTGGTTCTACTTGTGTTTACTCTAACGATTACTACTCTCAATGTCTTCCAGGTGCTGCTTCTTCTAGTTCTTCTTCTACTAGAGCTGCTTCTTCTACTACTTCTAGAGTTTCTCCAACTACTTCTAGAGTTCTTCTACTACTTCTAGATCTTCTTCTTCTACTACTACTAGAGTTCCACCAGTTGGTTCTGGTACTGCTACTTACTCTGGTAACCCATTTGTTGGTGTTACTCCATGGGCTAATGCATACTACGCTTCTGAAGTTTCTTCTTTGGCTATTCCATCTTTGACTGGTGCTATGGCTACTGCTGCTGCTGCTGTTGCTAAGGTTCCATCTTTTATGTGGTTGGATACTTTGGACAAGACTCCATTGATGGAACAAACTTTGGCTGATATTAGAACTGCTAACAAGAATGGTGGTAACTATGCTGGTCAATTTGTTGTTTATGATTTGCCAGATAGAGATTGTGCTGCTTTGGCTTCTAATGGTGAATACTCTATTGCTGATGGTGGTGTTGCTAAATACAAGAACTATATTGATACTATTAGACAAATTGTTGTTGAATACTCTGATATTAGAACTTTGTTGGTTATTGAACCAGATTCTTTGGCTAACTTGGTTACTAACTTGGGTACTCCAAAGTGTGCTAATGCTCAATCTGCTTACTTGGAATGTATTAACTACGCTGTTACTCAATTGAACTTGCCAAATGTTGCTATGTATTTGGATGCTGGTCATGCTGGTTGGTTGGGTTGGCCAGCTAACCAAGATCCAGCTGCTCAATTGTTTGCTAATGTTTACAAGAATGCCTCTTCTCCAAGAGCTTTGAGAGGTTTGGCTACTAATGTTGCTAACTACAACGGTTGGAACATTACTTCTCCACCATCTTACACTCAAGGTAACGCTGTTTACAACGAAAAGTTGTACATCCACGCTATTGGTCCATTGTTGGCTAACCACGGTTGGTCTAACGCTTTCTTCATTACTGATCAAGGTAGATCTGGTAAGCAACCAACTGGTCAACAACAATGGGGTGACTGGTGTAATGTTATTGGTACTGGTTTCGGTATTAGACCATCTGCTAACACTGGTGATTCTTTGTTGGATTCTTTTGTTTGGGTTAAGCCAGGTGGTGAATGTGACGGTACTTCTGACTCTTCTGCTCCAAGATTTGACTCTCACTGTGCTTTGCCAGATGCTTTGCAACCAGCTCCTCAAGCTGGTGCTTGGTTCCAAGCTTACTTTGTTCAATTGTTGACTAACGCTAACCCATCTTTTTTGTAA

**Beta-glucosidase gene sequence**

ATGCTGGTTGCTCATTCTTCATCCCCACACGCATCCCCAACACTTACCGCTTCTTTCGAAACTCCAGCCTATAGGTATTCCTCCTTCAGTTATACACAACTTACCTCAAATAGATATGCTACACCTTTGCCATCCCCAATATCATTTGCTACATCATTTGCTCCACCATTTTCTGAAGCATCTACTCTACTACCTGCTGGCGTGATATACACCACTTACTCATTAGAACCAACCACGACAACGGGAAGTGGTTCTGAGACTGAGTACGATCAGGAAGCCTATAATGAATTGTGGGCTAGTCTGTCCTACTCATCTGGACCACCTTTTACGACTACAGTTTCTCCAACTCCAGTTCCCACATCAGAGCTTGTTTTCCCACCTGCTTTGACACCATACTTGGCCAACCCAGAGAAAAAGTTACCAGAAAATTTCATCTGGGGTGTCGCTGGAAGTGCATGGCAAATAGAGGGTGGTTTGCAATTGGGTGGTAGAGGTCCATCAATACTGGATACTATTGGAGCTTTGCAATCTAACGACTCAGCTAGTGATGCCAATGTTGCTACTATGAACTATTATTTATACAAGCAGGATATTGCAAGACTTGCCGCATTGGGTGTACCATACTACTCTTTCTCAATCTCATGGTCTCGTATTGTTCCATTTGGAGTTGCAGGCTCTCCAGTTAATACGGAAGGTCTAGCTCACTACGATGACGTGATCAATACATGTCTGGAGTACGGAGTGATTCCTATAGTGACATTAGTTCATGCCGACTACCCTCCCAGTGTTGTAGCCGATATGGGTAACCTGACTTCTCATTACCTATATTACGCAAAGCAAGTTATGACGAGATATGCTGACAGAGTGCCTTACTGGGTGACTTTTAACGAACCTAACATTTCCGTGGGATCTGTAGCCAAGTCATACTCAATCTTGAAAGATATCCTATTGGCCCATGCTGCAGCTTACCACTGGTACAAAGAAACGCTTGGTGGTACAGCTCAAATTACTTTAAAGTTTGCAAACAATCTAGCCTTGCCATTGGATCCTACCAACTCCACACATACTACATCTGCACAGAGGTACCAAGCCTTTATTTTGGGTATTATGGGAAATCCCTTGTTATTGGGTGAGCAAATTCCTTCAGAGGTTCTATCTACCCCAAATATCACAATTGATGCTTTGACAGACGAGGAGTTAGCAACTGTTAAAGATACGGTAGATTTCATATCCATTGATCCTTACACGGCTCAATTCGCCTCTCCTGCTAGTGAAGGAATTGAAGCATGTGCCGCTAATTCCTCCAATCCATTGTGGCCATCTTGTGCCACATTGACTAACGTACAAGCTAACGGTTGGCTTATGGGAGAGGCTAGTAACGATTATGCATATATTGCACCCCAATACGTTCGTCAGCAGCTGGGTTACTTGTGGAATACTTTCAGACCCAAAGGTATCCTGATTTCTGAATATGGCTTCAACCCATTCGCAGATGCCAACCGAAGTCTAAACGCTCAGCGTTACGACCTGGAAAGGACTTTGTATTATCACTTTTTTTTGAGAGAAGTAATTACCGCCATCCACGAAGATGGCATCAATGTTATCGGTGCTCTAGCTTGGTCTGTTTTGGATAACGATGAGTTTGGTAGTTACGCTAATATGTATGGTCTACAGACAGTGAATAGGACAGACGGACGTTTTGAGAGGCAATATAAACGTTCTTTCTTTGACTACGTTGATTTTTTTTATCAGCACATTGCATCATAA

# Gene sequences of anchor protein are as follow:

**Sequences of anchor protein PIR1**

ATGGCTTACGCTCCAAAGGATCCATGGTCCACTCTGACTCCATCTGCTACTTACAAGGGTGGTATCACTGATTACTCTTCTACTTTTGGAATTGCTGTTGAACCAATTGCTACTACTGCTTCTTCTAAAGCTAAGAGAGCTGCTGCTATTTCCCAGATTGGTGACGGTCAAATCCAAGCTACTACTAAAACTACTGCTGCTGCCGTTTCTCAAATCGGAGATGGTCAAATTCAGGCTACTACTAAGACTAAGGCTGCTGCTGTTTCTCAAATTGGTGACGGACAAATTCAAGCTACTACCAAGACCACTTCTGCTAAGACTACTGCTGCCGCTGTTTCTCAAATTGGAGATGGTCAAATTCAAGCTACTACTAAGACTAAGGCCGCTGCTGTTAGTCAAATTGGAGATGGACAAATTCAGGCTACTACAAAGACTACTGCTGCTGCTGTGTCCCAGATTGGTGACGGACAAATTCAAGCCACTACTAAGACAACTGCTGCTGCTGTTTCACAAATTGGTGACGGTCAAATTCAAGCAACTACTAACACAACTGTTGCTCCAGTTTCCCAAATCACTGATGGACAGATTCAAGCCACAACTTTGACCTCTGCTACTATTATTCCATCTCCTGCTCCAGCTCCTATTACTAATGGTACTGACCCTGTTACTGCTGAAACTTGTAAATCTTCTGGTACTTTGGAAATGAACTTGAAGGGTGGTATCTTGACTGATGGTAAAGGTAGAATTGGATCTATTGTTGCCAACAGACAATTTCAGTTTGATGGACCACCACCTCAAGCTGGAGCTATCTACGCTGCTGGTTGGTCTATTACCCCAGAAGGTAACTTGGCTATTGGAGATCAAGATACTTTTTACCAATGTTTGTCTGGTAACTTTTATAACTTGTACGATGAACATATTGGTACTCAATGTAACGCTGTTCATTTGCAAGCTATTGATTTGTTGAACTGTTAA

**Sequences of anchor protein SED1**

ATGTTCTCTAACTCTACTTCTGCTTCTTCTACTGATGTTACTTCTTCTTCTTCCATTTCTACTTCTTCCGGTTCTGTTACTATTACCTCTTCCGAGGCTCCAGAATCTGATAATGGAACTTCTACTGCTGCTCCAACTGAAACTTCTACTGAGGCTCCAACAACTGCTATTCCTACTAATGGAACTTCCACTGAGGCTCCTACTGATACTACAACTGAAGCTCCAACTACTGCTTTACCAACTAACGGTACTTCTACTGAAGCTCCTACTGACACTACAACTGAGGCTCCAACTACTGCTTTGCCAACTAATGGTACCTCTACAGAGGCTCCAACTGATACTACTACTGAGGCTCCTACTACTGCTTTGCCAACTAATGGAACATCTACTGAAGCTCCAACTGATACCACTACTGAAGCCCCAACTACCGGATTGCCTACTAACGGAACTACTTCTGCTTTTCCACCAACTACTTCCCTTCCACCATCCAACACTACCACTACTCCACCATACAATCCATCAACTGATTATACTACTGATTACACCGTTGTTACTGAATACACTACTTACTGTCCTGAACCTACTACTTTTACTACCAACGGAAAGACTTATACCGTTACTGAGCCTACTACTTTGACTATTACTGATTGTCCATGTACAATTGAAAAACCAACTACTACTTCTACCACTGAATATACTGTTGTTACTGAATACACTACCTACTGTCCAGAACCAACTACTTTCACTACTAACGGTAAAACATACACAGTTACTGAACCAACTACCTTGACTATTACTGACTGTCCATGTACAATTGAAAAATCTGAAGCTCCTGAATCCTCTGTTCCAGTTACTGAATCAAAAGGTACTACTACTAAGGAAACTGGAGTTACTACTAAGCAAACTACTGCTAACCCATCTTTGACTGTTTCCACCGTTGTTCCTGTTTCTTCCTCTGCTTCTTCTCACTCTGTTGTGATTAACTCTAACGGTGCTAACGTTGTTGTTCCAGGTGCTTTGGGTTTGGCTGGTGTCGCTATGTTGTTCTTGTAA

**Sequences of anchor protein AGA1**

ATGATTAATATTAATGATATTACTTTCTCTAACCTTGAAATTACTCCATTGACTGCTAACAAGCAACCTGATCAAGGATGGACTGCAACTTTTGATTTTAGTATTGCTGATGCTTCTTCTATTAGAGAAGGTGACGAGTTTACTCTTAGTATGCCTCATGTTTACAGAATTAAGTTACTTAACTCCTCCCAAACTGCTACTATTTCATTGGCTGATGGTACAGAAGCCTTTAAATGTTATGTCTCCCAACAAGCAGCCTACCTGTACGAAAACACCACTTTTACTTGTACCGCTCAAAACGATCTTTCTTCCTACAATACTATTGATGGTTCTATTACTTTTTCTTTAAACTTTAGTGATGGTGGTTCCTCTTATGAATATGAATTGGAAAATGCTAAGTTTTTCAAGTCTGGTCCAATGCTTGTTAAATTGGGTAACCAAATGTCTGATGTTGTTAACTTTGATCCTGCTGCCTTTACTGAAAACGTCTTTCATAGTGGTAGATCTACTGGTTACGGTAGTTTTGAGTCTTACCATTTGGGTATGTACTGTCCTAACGGTTACTTCTTGGGAGGTACTGAGAAAATTGATTACGACAGTTCTAATAATAACGTTGATTTGGATTGTTCTTCTGTGCAGGTTTATAGTTCCAATGATTTTAACGACTGGTGGTTTCCTCAGTCATATAACGATACTAACGCTGATGTAACATGTTTTGGATCAAACTTGTGGATTACTCTAGATGAAAAATTGTACGATGGAGAAATGTTGTGGGTTAATGCTTTGCAATCTTTGCCTGCTAACGTTAATACTATTGACCATGCTTTGGAGTTCCAATACACCTGTCTTGACACAATTGCTAACACTACTTATGCTACACAATTCTCTACTACTAGAGAGTTTATCGTTTACCAAGGAAGGAATTTGGGTACTGCTTCTGCTAAGTCCTCTTTTATTAGTACTACAACTACTGATTTGACTTCTATTAATACTTCGGCTTACTCTACTGGTTCTATTTCTACAGTTGAAACTGGTAATAGAACAACTTCTGAAGTTATTAGTCATGTTGTTACCACTTCTACAAAATTGTCTCCTACAGCTACTACTTCTTTGACAATCGCTCAAACTTCTATCTACAGTACAGATTCAAACATCACTGTTGGTACCGACATTCACACAACTTCTGAAGTCATAAGTGATGTTGAAACTATTTCTAGAGAAACAGCTTCTACTGTTGTTGCTGCTCCAACTTCTACTACCGGTTGGACTGGTGCTATGAACACTTACATTTCTCAGTTCACTTCTTCATCTTTCGCTACTATAAACTCAACACCAATTATTAGTTCATCTGCTGTTTTTGAAACTTCCGATGCTTCAATAGTTAATGTTCATACTGAGAATATTACAAACACTGCAGCTGTTCCTTCTGAGGAACCTACATTCGTTAACGCCACTAGAAATAGTTTGAACTCTTTTTGCTCATCCAAGCAACCATCATCCCCATCTTCCTATACTTCTTCACCTTTGGTTTCCTCCTTGTCCGTTTCCAAGACTTTGTTGTCTACTTCTTTCACTCCATCTGTTCCTACTTCTAATACTTACATTAAGACTAAGAACACTGGTTACTTTGAACATACTGCCTTGACCACTTCATCTGTTGGTCTAAACAGTTTCTCTGAAACCGCTGTTTCATCCCAAGGTACTAAGATTGATACCTTTTTGGTTTCTTCTTTGATTGCTTACCCATCTTCCGCTTCTGGATCTCAATTGTCTGGTATTCAGCAAAACTTTACCTCCACTTCTTTGATGATTTCTACTTATGAGGGTAAAGCTAGTATTTTCTTTAGTGCTGAATTGGGATCTATTATTTTCTTGTTGCTGAGTTACTTGTTGTTTTAA
